# How does a Social and Behavioral Change Communication Intervention Predict Menstrual Health and Hygiene Management: A Cross-Sectional Study

**DOI:** 10.1186/s12889-019-7359-z

**Published:** 2019-08-02

**Authors:** Astha Ramaiya, Alka Malhotra, Carmen Cronin, Sarah Stevens, Kelli Kostizak, Animesh Sharma, Shailesh Nagar, Suruchi Sood

**Affiliations:** 1 Bloomberg School of Public Health, Johns Hopkins University, 615 N. Wolfe Street, Baltimore, MD 25880 USA; 2 UNICEF, New Delhi, India; 3 Department of Community Health and Prevention, Drexel University, Philadelphia, PA USA; 4 NR Management Consultants, New Delhi, India

**Keywords:** SBCC, menstrual health and hygiene management, menstrual hygiene management, adolescent girls, India

## Abstract

**Background:**

Interventions in India to improve menstrual health and hygiene management (MHHM) have been implemented at the national, state, district and school level. However, evaluations of these interventions have been scarce. The objective of the study was to determine if a social and behavioral change communication (SBCC) intervention (*GARIMA)* had a relationship with knowledge, attitudes, interpersonal communication, restrictions and MHHM using a comparison group post-test only design among 2206 adolescent girls.

**Methods:**

Intervention villages and adolescent girls were selected through stratified random sampling based on where *GARIMA* was implemented. Villages and adolescent girls in comparison villages were matched socio-demographically to intervention villages and adolescent girls. Multi-level logistic regressions assessed the relationship between the encoded exposure, mediators and MHHM.

**Results:**

The results showed that the encoded exposure predicted all behaviors corresponding to MHHM. Additionally, adolescent girls in the high encoded exposure group had significantly higher knowledge about puberty and reproductive parts (AOR: 2.03 (95% CI: 1.31 – 3.15)), positive attitudes towards gender (AOR: 1.48 (95% CI: 1.02 – 2.16)) and higher levels of some discussion and dialogue (AOR: 1.41 (95% CI: 1.04 – 1.92)).

**Conclusions:**

Future programs should use SBCC to improve MHHM behavior but involve families, peers and community members to a greater extent in order to improve attitudes towards menstruation, attitudes towards restrictions, attitudes towards absorbent use and reduce restrictions within the community.

## Background

Within the last decade, menstrual health and hygiene management (MHHM) has been considered a public health issue because of its link to health, social justice and human rights [1, 2]. MHHM is defined as the “access and use of private toilet/bathroom with soap and water to wash hands and have a bath during menstruation and use of clean menstrual management material to absorb or collect blood that is stored in a safe, clean place and changed at least three times a day for the duration of the menstruation period and accessing facilities to ultimately dispose of used menstrual management materials” [3].

MHHM has evolved into a public health priority to decrease gender inequality in education, especially among adolescent girls. Commentaries within scientific literature and global organizations have called to increase both knowledge and social support around menarche and MHHM behaviors worldwide [4–6]. Literature from around the world documents that adolescent girls feel ashamed once they start menstruation [4]. Because of this shame, adolescent girls have lower self-confidence and ability to make decisions regarding their personal sexual and reproductive health [4]. In particular, lack of adequate water and sanitation facilities in school within South Asia affects the dignity of adolescent girls to meet their MHHM needs [7–9].

Interventions in India to improve MHHM have been implemented at the national, state, district and school level [8, 10–17]. The interventions address the lack of access to menstrual hygiene products [8, 10, 14] and information [12–17]. The Menstrual Hygiene Scheme had an objective of decreasing the cost of sanitary pads to Rs 6 ($0.10) for a pack of 6 pads across 107 districts and seven states [10, 11]. After 2014, the individual states procured and distributed the sanitary pads in rural areas. Health workers were charged with distributing sanitary pads and to organize monthly meetings at health centers for adolescent girls to talk about menstrual hygiene. Another Government of India flagship program is the SABLA program which promotes awareness about health, hygiene, nutrition, adolescent reproductive & sexual health, and family/child care, in particular among out-of-school adolescent girls [14]. The Swach Bharat Mission is a campaign to improve efforts to achieve universal sanitation coverage across India [18]. The guidelines for the Swach Bharat Mission include the need to provide knowledge about menstruation and have hygiene and sanitation linked to the menstrual cycle. The guideline also proposes implementing safe disposal facilities for menstrual waste [19].

To support the national interventions, smaller state initiatives have been conducted [12, 14]. The three areas that programs in this category have addressed adequate MHHM include: 1) building capacities/ supporting flagship programs, 2) promoting hygiene and 3) outreach [14]. In addition to national- and state-based interventions, other smaller initiatives have been implemented either in districts or in schools focusing on information provision to change behavior [15–17].

To the authors’ knowledge, of the various interventions found in the Indian literature, only five of them have evaluated their effectiveness on adequate MHHM [8, 12, 15–17]. However, very few evaluations have been conducted with a comparison group, which compromises both validity and reliability of the evaluation; the studies also lack power analysis to determine effect size; exposure has been coded dichotomously (exposed versus unexposed) and there is no mention of theory or conceptual framework when designing and evaluating the interventions [8, 12, 15, 17].

The objective of this research is to understand if encoded exposure to a social and behavioral change communication (SBCC) intervention (*GARIMA),* comprising of dose and recall has an effect on knowledge, attitudes, interpersonal communication, restrictions and MHHM. It is hypothesized that *GARIMA* will improve knowledge, attitudes, interpersonal communication and MHHM behaviors and reduce restrictions in the communities.

### Intervention Overview

Scientific literature demonstrates that SBCC campaigns play a necessary role in adoption and retention of individual health behavior and social change [20]. *GARIMA* was a SBCC community-based communication program designed and funded by UNICEF to make MHHM a normal experience for thousands of adolescent girls using audio-visual, print media and life skills-based activities. A communication package was implemented with local NGO which included girl groups watching films, reading a storybook, getting a personal diary, a poster and an apron. Monthly adolescent girls group meetings were facilitated by a peer educator; one-on-one discussions with peer educators and frontline health workers; life skills-based activities by peer educators; monthly mothers’ group meetings and small group meetings with fathers [21]. Additionally, there was capacity building of the implementers (frontline health workers, and peer educators) through discussions about MHHM [21]. *GARIMA* was a ground-up intervention where adolescent girls could choose which topics, they wanted to discuss for their group meetings based on local customs and contexts.

The intervention occurred in three districts (Mirzapur, Jaunpur and Sonebhadra) of Uttar Pradesh with local partners reaching approximately 64,000 adolescent girls across 1975 villages. These districts were chosen by UNICEF and local partners purposively based on the location and MHHM indicators deduced during the baseline [22]. The intervention occurred between 2013 – 2016. The first phase of the intervention took place between 2013 – 2016 in Mirzapur. Thereafter, the intervention expanded to Jaunpur and Sonebhadra from 2014 – 2016 based on the lessons learnt from Mirzapur [23].

The goal of *GARIMA* was for adolescent girls to break the culture of silence around menstruation by initiating dialogue, sustaining conversations with peers, family and community and thereby influencing change to normalize menstruation. It was hypothesized, by breaking the culture of silence, adolescent girls could manage their menstruation in a healthy and hygienic manner [13].

## Methods

### Theoretical Approach

The *GARIMA* program evaluation was examined within the lens of the ideation framework, which specifically examines the effects of communication interventions on individual and social change [24]. *GARIMA* aimed to improve adolescent girls’ knowledge, attitudes, interpersonal communication and reduce restrictions, in order to facilitate better MHHM.

### Study Design & Setting

The *GARIMA* evaluation utilized a post-test only case-comparison design to examine the effectiveness of the intervention, specifically dose and recall on MHHM between December 2017 and January 2018. This design was chosen because there was a lack of overlap in villages selected at baseline and subsequently for the intervention (*n*=12).

### Sampling Frame and Participants

The sampling was a combination of purposive and random sampling. Selection of the districts and the villages where the intervention occurred was purposive. However, selection of the participants was random. The sampling first occurred at the village level and then at the adolescent girl level outlined in Appendix 1 and Appendix 2*.* In summary, there were a total of 240 villages sampled (120 intervention and comparison, each) and 2206 adolescent girls (1132 in intervention villages and 1074 in comparison villages) who were 12-18 years and had initiated menstruation. Comparison villages and adolescent girls were matched demographically with intervention villages and adolescent girls.

### Data Collection

A structured questionnaire was translated into Hindi and pre-tested. The questionnaire had thirteen sections. Trained data collectors used computer-assisted personal interviewing was used to complete the questionnaires to reduce interviewer bias and improve inter-rater reliability.

### Variables

There were five main dependent variables: preparation of clean absorbent, storage of clean absorbent, frequency of changing, disposal and hygiene [3]. All five behaviors were modeled as categorical variables. Validity testing of the MHHM scale demonstrated five different constructs [3].

The intervention is being operationalized by the encoded exposure (dose and recall) rather than exposure (intervention versus comparison villages) only, in order to understand the intervention as a gradient. Since the intervention was completed in 2016 and not all adolescent girls (aged 10 -19) in the intervention villages were part of *GARIMA* throughout the three years. The encoded exposure variable was operationalized by creating a variable to measure dose and recall of *GARIMA* among adolescent girls in intervention villages and reduce recall bias. An additive scale of 10 items ranging from 0-47 was created, taking into account dose and recall. Four ordinal categories were created: 1) none for the adolescent girls in the comparison villages (score of 0), 2) low (score of 4 – 18), 3) medium (score of 19 – 24) and 4) high (score of 25 – 47).

The conceptual model had both mediators and confounders. The mediators included knowledge, attitudes, interpersonal communication and restrictions. Knowledge was made into three sub-scales (knowledge about: puberty, reproductive organs and absorbent use); attitudes were made into four sub-scales (attitudes towards: menstruation, absorbent use, restrictions and gender); interpersonal communication was kept as a single variable and restrictions were made into seven sub-scales (clothing, food, mobility, social/religious, personal, social and structural) *(Table* *1**)*. Since all additive indexes for the mediators were not normally distributed, categories were created based on the distribution of the data. Confounders included socio-demographic, socio-economic characteristics and duration since menarche.

**Table 1:** Operationalization of Mediators

Independent variables # of sub-measures	Sub-Scales	# of questions in Sub-scale	Range of Sub-Scale	Final labels
Knowledge [3]	Knowledge about puberty	2	0-13	Low: 0-4
				Medium: 5 - 8
				High: 9 - 13
	Knowledge about reproductive organs	6	0-12	Low: 0
				Medium: 1- 11
				High: 12
	Knowledge about absorbent use	10	4-18	Low: 0 - 8
				Medium: 9 - 13
				High: 14 - 18
Attitudes [4]	Attitudes towards menstruation	6	0-24	Negative: 0 – 12
				Neutral: 13 – 15
				Positive: 16 - 24
	Attitudes towards absorbent use	10	0-40	Negative: 0 – 21
				Neutral: 22 – 28
				Positive: 29 - 40
	Attitudes towards restrictions	4	0-20	Negative: 0 – 7
				Neutral: 8 – 11
				Positive: 12 - 20
	Attitudes towards gender	5	0-20	Negative: 0 – 11
				Neutral: 12 – 15
				Positive: 16 - 20
Interpersonal Communication [1]		9	1-18	0: No discussion
				1 – 17: Some discussion/ dialogue
				18: All discussion
Restrictions [7]	Clothing	6	0-6	0: Present
				1: Not present
	Food	6	0-6	0 : Present
				1: Not present
	Mobility	5	0-5	0:Present
				1: Not present
	Social/Religious	4	0-4	0: Present
				1: Not present
	Personal	21	0-12	0: Present
				1: Not present
	Social	21	0-11	0: Present
				1: Not present
	Structural	21	0-7	0: Present
				1: Not present

### Sample Size Calculation

A sample size calculation was conducted using Valente’s differences method, taking into account the design effect of 1; a 5% difference in adequate MHHM pre and post intervention, α of 0.05 and a β of 0.8 [25]. This formula gave a sample size of 400 AG per district in the intervention villages and 400 AG per district in the comparison villages, giving a total of 2400 adolescent girls (pre and post menarche) across three districts in the intervention and comparison villages.

### Analysis

Multilevel logistic regressions were conducted to understand: 1) the effect of the encoded exposure on individual mediators and 2) the effect of the encoded exposure and mediators on behaviors comprising of MHHM. District, block and village were modelled as random effects. Religion and marital status were dropped from the regression because almost all adolescent girls were Hindu and unmarried. Adjusted Odds Ratios (AOR) were reported with 95% confidence intervals (95% CI). If the 95% CI included 1, the predictor was not considered significant. All analyses were conducted on Stata 14.0 [26]. Since the proportion of missing values was minimal (<2%), missing values were excluded from the analysis.

## Results

In terms of socio-demographic characteristics of the population: overall, there was an equal proportion of adolescent girls from Jaunpur (36.7%), Sonebhadra (29.8%) and Mirzapur (33.5%). Ninety seven percent of the adolescent girls were Hindu; more than 90% were scheduled caste/tribe or other backward caste; 47% lived in temporary housing and 99% were unmarried [3]. Forty eight percent of the adolescent girls were in the comparison villages and 15.96%, 21.62% and 13.74% of the adolescent girls were in the low, medium and high exposure, respectively. In terms of MHHM behaviors, overall, 23.39% of adolescent girls adequately prepared their clean absorbent; 60% adequately stored their clean absorbent; 63.83% changed their absorbent three or more times a day; 40.53% adequately disposed of their used menstrual material and 47.74% adequately practiced personal and menstrual hygiene. Table 2 shows significant differences between the different levels of the encoded exposure for district, religion, caste, type of house, preparation of clean absorbent, storage of clean absorbent, frequency of changing, disposal and hygiene.

**Table 2:** Socio-demographic, Socio-economic Characteristics and MHHM behaviors among Adolescent Girls in Rural Uttar Pradesh (*n*=2206)

N		Encoded Exposure					
		Overall	None	Low	Medium	High	*p-value*
		2206	1074	352	477	303	
District	*Jaunpur*	36.76%	35.75%	36.93%	37.53%	38.94%	0.002
	*Sonebhadra*	29.74%	30.82%	34.09%	30.40%	19.80%	
	*Mirzapur*	33.50%	33.43%	28.98%	32.08%	41.25%	
Religion	*Non-Hindu*	2.95%	2.61%	2.27%	2.31%	5.94%	0.01
	*Hindu*	97.05%	97.39%	97.73%	97.69%	94.06%	
Caste	*General Caste*	9.38%	8.66%	9.66%	8.81%	12.54%	0.02
	*Scheduled Caste*	50.54%	47.77%	53.98%	54.93%	49.50%	
	*Other Backward Caste*	40.07%	43.58%	36.36%	36.27%	37.95%	
Age (years)		16.24	16.25	16.15	16.22	16.34	0.58
Education (grade)		9.13	9.42	9.25	10.0	9.85	0.25
Type of house	*Kutcha*	47.05%	42.74%	54.26%	53.25%	44.22%	<0.01
	*Semi-pucca*	24.52%	25.70%	21.02%	23.06%	26.73%	
	*Pucca*	28.42%	31.56%	24.72%	23.69%	29.04%	
Marital Status	*Married*	0.86%	0.74%	1.14%	1.05%	0.66%	0.86
	*Unmarried*	98.87%	98.88%	98.58%	98.95%	99.01%	
	*Other*	0.27%	0.37%	0.28%	0%	0.33%	
Duration since menarche	*< 1 year*	13.65%	13.57%	14.45%	12.34%	15.10%	0.70
	≥*1 year*	86.35%	86.43%	85.55%	87.66%	84.90%	
Preparation of clean absorbent	*Inadequate (inad)*	52.18%	59.12%	51.42%	43.82%	41.58%	< 0.01
	*Semi-adequate (semiad)*	24.43%	18.99%	23.86%	32.49%	31.68%	
	*Adequate (ad)*	23.39%	21.88%	24.72%	23.69%	26.73%	
Storage of clean absorbent	*inad*	22.71%	30.54%	14.77%	16.77%	13.53%	<0.01
	*semiad*	16.77%	19.37%	15.06%	14.05%	13.86%	
	*ad*	60.52%	50.09%	70.17%	69.18%	72.61%	
Frequency of changing	*inad*	36.17%	52.42%	26.70%	15.30%	22.44%	<0.01
	*ad*	63.83%	47.58%	73.30%	84.70%	77.56%	
Disposal	*inad*	34.72%	44.23%	28.41%	24.74%	24.09%	<0.01
	*semiad*	24.75%	24.12%	24.43%	25.58%	26.07%	
	*ad*	40.53%	31.66%	47.16%	49.69%	49.83%	
Hygiene	*inad*	36.40%	42.55%	31.25%	28.51%	33.0%	<0.01
	*semiad*	15.87%	15.08%	19.32%	17.40%	12.21%	
	*ad*	47.74%	42.36%	49.43%	54.09%	54.79%	

### Relationship between Encoded Exposure and Mediators

Table 3 shows the multivariable analysis examining the relationship between the encoded exposure and mediators. Knowledge about puberty, knowledge about reproductive parts, attitudes towards gender and interpersonal communication was significantly higher in intervention villages versus comparison villages.

**Table 3:** Multi-level Multivariable Model Assessing the Relationship between the Encoded Exposure and Mediators (*n*=2167)^b^

Characteristics	High encoded exposure AOR (95% CI)
Knowledge about puberty	
*Low*	1.0
*Medium*	1.49 (1.12 – 1.99)^**^
*High*	2.97 (1.89 – 4.65)^***^
Knowledge about reproductive parts	
*Low*	1.0
*Medium*	1.87 (1.36 – 2.57)^***^
*High*	2.04 (1.32 – 3.17)^**^
Attitudes towards gender	
*Negative*	1.0
*Neutral*	1.10 (0.77 – 1.58)
*Positive*	1.47 (1.01 – 2.14)^*^
Interpersonal communication	
*No discussion*	1.0
*Some discussion and dialogue*	1.40 (1.03 – 1.90)^*^
*All dialogue*	0.93 (0.55 – 1.57)
Random effects	
*District (var)*	1.75 e^-33^
*District>Block (var)*	32.10 (14.17 – 72.66)
*District>Block>Village (var)*	0.87 (0.54 – 1.42)^***^

*: Significant difference between independent variable and MHHM behavior at *p****≤***0.05 level.

**: Significant difference between independent variable and MHHM behavior at *p****≤***0.01 level.

***: Significant difference between independent variable and MHHM behavior at *p****≤***0.001 level.

b: Controlled for socio-demographics, socio-economic characteristics, duration since menarche, knowledge about puberty, knowledge about reproductive parts, knowledge about absorbent use, attitudes towards menstruation, attitudes towards absorbent use, attitudes towards restrictions, attitudes towards gender, interpersonal communication, personal restrictions, social restrictions, structural restrictions and random effects for district, block and village

After controlling for all other predictors: adolescent girls in the high encoded exposure group had greater odds of having medium (AOR: 1.49 (95% CI: 1.12 – 1.99)) and high knowledge about puberty (AOR: 2.97 (95% CI: 1.89 – 4.65)), respectively, in comparison to adolescent girls with low knowledge about puberty, in each village. Additionally, being in the high encoded exposure group increased the odds of having medium (AOR: 1.87 (95% CI: 1.36 – 2.57)) and high knowledge (AOR: 2.04 (95% CI: 1.32 – 3.17)) about reproductive parts in comparison to adolescent girls with low knowledge about reproductive parts, respectively, in each village.

Adolescent girls with high encoded exposure had a significantly greater odds of positive attitudes towards gender (AOR: 1.47 (95% CI: 1.01 – 2.14)), in comparison to adolescent girls with negative attitudes towards gender.

Adolescent girls in the high encoded exposure group had greater odds of having some discussion and some dialogue (AOR: 1.40 (95% CI: 1.03 – 1.90)) in comparison to adolescent girls who had no discussion, in each village.

Lastly, random effects for district, block and village was significant indicating that the results were generalizable to each village.

All other mediators did not have a significant relationship with the exposure.

### Relationship between Encoded Exposure, Mediators and MHHM Behaviors

Table 4 is the multivariable analysis examining the relationship between the encoded exposure, mediators and MHHM.

**Table 4:** Multi-level Multivariable Model Assessing the Relationship between the Encoded Exposure, Mediators and MHHM (*n*=2167)^a^

Characteristics	Adequate preparation of clean absorbent AOR (95% CI)	Adequate storage of clean absorbent AOR (95% CI)	Adequate frequency of changing AOR (95% CI)	Adequate disposal AOR (95% CI)	Adequate hygiene AOR (95% CI)
Encoded Exposure					
*None*	1.0	1.0	1.0	1.0	1.0
*Low*	1.23 (0.92 – 1.66)	2.14 (1.50 – 3.05)***	2.64 (1.71 – 4.07)***	1.73 (1.22 – 2.43)**	1.67 (1.16 – 2.39)**
*Medium*	1.38 (1.04 – 1.84)*	1.91 (1.35 – 2.70)***	4.87 (3.10 – 7.63)***	1.79 (1.28 – 2.50)**	1.78 (1.25 – 2.55)**
*High*	1.36 (0.98 – 1.88)	2.10 (1.42 – 3.12)***	2.52 (1.57 – 4.04)***	1.93 (1.33 – 2.80)**	1.41 (0.95 – 2.10)
Knowledge about puberty					
*Low*	1.0	1.0	1.0	1.0	1.0
*Medium*	1.14 (0.92 – 1.42)	0.78 (0.62 – 0.98)*	1.25 (0.96 – 1.61)	0.88 (0.71 – 1.09)	1.14 (0.91 – 1.43)
*High*	1.17 (0.81 – 1.70)	1.08 (0.70 – 1.66)	2.21 (1.30 – 3.76)**	0.78 (0.54 – 1.13)	1.31 (0.87 – 1.96)
Knowledge about reproductive parts					
*Low*	1.0	1.0	1.0	1.0	1.0
*Medium*	0.89 (0.71 – 1.10)	1.25 (1.0004 – 1.56)*	1.18 (0.92 – 1.51)	1.50 (1.21 – 1.85)***	1.02 (0.82 – 1.27)
*High*	0.92 (0.66 – 1.28)	1.87 (1.29 – 2.71)**	0.95 (0.61 – 1.47)	1.56 (1.12 – 2.18)*	1.46 (1.01 – 2.12)*
Attitudes towards gender					
*Negative*	1.0	1.0	1.0	1.0	1.0
*Neutral*	1.40 (1.08 – 1.80)**	1.17 (0.91 – 1.51)	0.85 (0.64 – 1.14)	1.12 (0.88 – 1.43)	0.88 (0.69 – 1.14)
*Positive*	1.57 (1.21 – 2.05)***	1.61 (1.22 – 2.12)***	0.94 (0.69 – 1.28)	1.00 (0.78 – 1.29)	1.02 (0.78 – 1.33)
Interpersonal communication					
*No discussion*	1.0	1.0	1.0	1.0	1.0
*Some discussion and dialogue*	1.09 (0.88 – 1.35)	1.42 (1.14 – 1.77)**	1.31 (1.03 – 1.67)*	1.38 (1.12 – 1.70)**	1.39 (1.12 – 1.72)**
*All dialogue*	1.28 (0.89 – 1.84)	0.95 (0.67 – 1.35)	0.91 (0.59 – 1.40)	0.89 (0.63 – 1.26)	1.87 (1.26 – 2.77)**
Random Effects					
*District (var)*	0.0072 (0.00016 – 0.32)	1.02 e ^-36^	0.23 (0.06 – 0.84)	0.05 (0.005 – 0.40)	3.36 e ^-34^
*District>Block (var)*	8.38 e ^-33^	0.038 (0.007 – 0.21)	0.28 (0.14 – 0.56)	0.04 (0.005 – 0.29)	0.04 (0.0027 – 0.55)
*District>Block>Village (var)*	0.26 (0.15 – 0.44)***	0.20 (0.10 – 0.42)***	0.56 (0.40 – 0.79)***	0.24 (0.13 – 0.43)***	0.38 (0.23 – 0.62)***

*: Significant difference between independent variable and MHHM behavior at *p****≤***0.05 level.

**: Significant difference between independent variable and MHHM behavior at *p****≤***0.01 level.

***: Significant difference between independent variable and MHHM behavior at *p****≤***0.001 level.

a: Controlled for socio-demographics, socio-economic characteristics, duration since menarche, knowledge about puberty, knowledge about reproductive parts, knowledge about absorbent use, attitudes towards menstruation, attitudes towards absorbent use, attitudes towards restrictions, attitudes towards gender, interpersonal communication, personal restrictions, social restrictions, structural restrictions and random effects for district, block and village

#### Predictors of adequate preparation of clean absorbent

After controlling for all other predictors: Adolescent girls who had medium exposure had a 1.38 (95% CI: 1.04 – 1.84) times greater odds of preparing the clean absorbent versus adolescent girls who had no exposure, in each village. Adolescent girls who had low and high exposure were not significantly different in terms of preparing the clean absorbent from adolescent girls who had no exposure.

Mediating predictors which were significantly associated with the encoded exposure and adequate preparation of clean absorbent were attitudes towards gender. Adolescent girls who had neutral and positive attitudes versus negative attitudes towards gender had a 1.40 (95% CI: 1.08 – 1.80) and 1.57 times (95% CI: 1.21 – 2.05) greater odds of practicing adequate preparation of clean absorbent, respectively, in each village.

Other mediating predictors which were significantly associated with adequate preparation of clean absorbent were positive attitudes towards absorbent use, neutral attitudes towards restrictions, not having food restrictions, not having personal restrictions and not having structural restrictions (not shown on table).

Random effects for district, block and village was significant indicating that the results were generalizable to each village.

#### Predictors of adequate storage of clean absorbent

Adolescent girls who had low, medium and high exposure had a 2.14 (95% CI: 1.50 – 3.05), 1.91 (95% CI: 1.35 – 2.70) and 2.10 (95% CI: 1.42 – 3.12) times greater odds of having adequate storage of clean absorbent, respectively in comparison to adolescent girls who had no exposure, in each village.

Mediating predictors which were significantly associated with the encoded exposure and adequate storage of clean absorbent were knowledge about puberty, knowledge about reproductive parts, attitudes towards gender and interpersonal communication. After controlling for all other predictors: adolescent girls who had medium knowledge about puberty had 22% lower odds of practicing adequate storage of clean absorbent (95% CI: 2% - 38%) in comparison to adolescent girls who had low knowledge about puberty, in each village. Adolescent girls who had medium and high knowledge about reproductive parts had a 1.25 (95% CI: 1.0004 – 1.56) and 1.77 (95% CI: 1.23 – 2.56) times greater odds of adequate storage of clean absorbent in comparison to those who had low knowledge about reproductive parts, in each village, respectively. Adolescent girls who had positive attitudes towards gender had a 1.61 (95% CI: 1.22 – 2.12) times greater odds of adequate storage of clean absorbent in comparison to those who had negative attitudes towards gender, in each village. Adolescent girls who had some discussion and dialogue versus no discussion had 42% greater odds (95% CI: 14% – 77%) of practicing adequate storage of clean absorbent, in each village.

Other mediating predictors significantly associated with adequate storage of clean absorbent were medium and high knowledge about absorbent use, neutral attitudes towards restrictions and not having social/religious restrictions (not shown on table).

Random effects for district, block and village was significant indicating that the results were generalizable to each village.

#### Predictors of adequate frequency of changing

Adolescent girls who had low, medium and high encoded exposure had a 2.64 (95% CI: 1.71 – 4.07), 4.87 (95% CI: 3.10 – 7.63) and 2.52 (95% CI: 1.57 – 4.04) times greater odds of changing the absorbent three or more times a day, respectively in comparison to adolescent girls who had no exposure, in each village.

Mediating predictors associated with the encoded exposure and adequate frequency of changing were knowledge about puberty and interpersonal communication. Adolescent girls who had high knowledge about puberty had 2.21 times greater odds (95% CI: 1.30 – 3.76) of practicing adequate frequency of changing versus those who had low knowledge about puberty, in each village. Adolescent girls who had some discussion and some dialogue versus no discussion, had 31% greater odds (95% CI: 3% – 67%) of practicing adequate frequency of changing, in each village.

Other mediators which were associated with adequate frequency of changing were medium and high knowledge about absorbent use and no personal restrictions (not shown on table).

Random effects for district, block and village was significant indicating that the results were generalizable to each village.

#### Predictors of adequate disposal

Adolescent girls who had low, medium and high exposure, had a 1.73 (95% CI: 1.22 – 2.43), 1.79 (95% CI: 1.28 – 2.50) and 1.93 (95% CI: 1.33 – 2.80) times greater odds of practicing adequate disposal, respectively in comparison to adolescent girls who had no exposure, in each village.

Mediating predictors associated with the encoded exposure and adequate disposal included knowledge about reproductive parts and interpersonal communication. After controlling for all other predictors: Adolescent girls with medium and high knowledge about reproductive parts had 1.50 (95% CI: 1.21 – 1.85) and 1.56 (95% CI: 1.12 – 2.18) times greater odds of practicing adequate disposal, respectively in comparison to adolescent girls with low knowledge about reproductive parts, in each village. Adolescent girls who had some discussion and some dialogue had a 1.38 (95% CI: 1.12 – 1.70) times greater odds of adequate disposal in comparison to adolescent girls who had no discussion, in each village.

Other mediators which were associated with adequate disposal included medium and high knowledge about absorbent use, positive attitudes towards menstruation, neutral and positive attitudes towards restrictions and no structural restrictions.

Random effects for district, block and village was significant indicating that the results were generalizable to each village.

#### Predictors of adequate hygiene

Adolescent girls in the low and medium exposure in comparison to none, had a 1.67 (95% CI: 1.16 – 2.39) and 1.78 (95% CI: 1.25 – 2.55) times greater odds of practicing adequate hygiene, respectively, in each village. Adolescent girls who had high exposure were not significantly different in terms of adequate hygiene in comparison to adolescent girls who had no exposure.

Mediating predictors associated with the encoded exposure and adequate hygiene were knowledge about reproductive parts and interpersonal communication. After controlling for all other predictors: Adolescent girls with high knowledge about reproductive parts versus low knowledge about reproductive parts, had a 1.46 times (95% CI: 1.01 – 2.12) greater odds of practicing adequate hygiene, in each village. Adolescent girls who had some discussion and dialogue and all dialogue, had a 1.39 (95% CI: 1.12 – 1.72) and 1.87 (95% CI: 1.26 – 2.77) times greater odds of practicing adequate hygiene, respectively, in comparison to adolescent girls who had no discussion, in each village.

Random effects for district, block and village was significant indicating that the results were generalizable to each village.

## Discussion

The objective of this research was to understand if encoded exposure to a SBCC intervention (*GARIMA),* comprising of dose and recall had an effect on knowledge, attitudes, interpersonal communication, restrictions and MHHM. The results showed that adolescent girls in the high encoded exposure group had higher knowledge about puberty, higher knowledge about reproductive parts, positive attitudes towards gender and significantly higher levels of some discussion and dialogue. The mediators which were associated with encoded exposure also predicted all behaviors corresponding to MHHM. Significant mediators which had a relationship with three out of the five behaviors corresponding to MHHM included knowledge, attitudes, interpersonal communication and restrictions.

This study used a new method to operationalize the intervention using an encoded exposure variable (operationalized through dose and recall) versus a dichotomous variable (exposed versus unexposed). Dose and recall were made into a composite and then made into an ordinal variable. This method has been used in other analyses of SBCC programs in India and Mozambique to understand the effect of the intervention as a gradient [27, 28]. Within the realm of MHHM, only one evaluation included a comparison group and the intervention was operationalized dichotomously (exposed district versus unexposed district) [16]. The results of this study demonstrate that there are differences is behavior proportions between the different levels of the intervention. Therefore, it is recommended that in the future, interventions take a dose and recall approach to measuring exposure in order to understand the gradient of the intervention.

*GARIMA* was a SBCC intervention which involved girl groups watching films, reading a storybook, getting a personal diary, a poster and apron. The results demonstrate that adolescent girls with higher levels of the encoded exposure had adequate behaviors comprising of MHHM. Additionally, adolescent girls who had higher encoded exposure had higher knowledge about puberty, higher knowledge about reproductive parts, positive attitudes towards gender and increased interpersonal communication about menstruation. Since the goal of *GARIMA* was to break the culture of silence, the intervention succeeded in achieving its key objectives. Information based interventions have been conducted in India and showed an improvement in awareness and behavior [12, 15, 17]. A program in Maharashtra evaluated the effectiveness of a pre-tested handmade flipbook and showed better awareness about menstruation, more adolescent girls in community-based organizations, increase in readymade sanitary pad users and increased drying in the sun for adolescent girls who used cloth [12]. Two of the interventions implemented an education program in school and demonstrated an increase in knowledge and MHHM behaviors [15, 17]. The study results also demonstrated that knowledge about absorbent use, attitudes towards menstruation, attitudes towards absorbent use, attitudes towards restrictions and all restrictions did not vary with the levels of the encoded exposure.

Implications of the study at the national level are noteworthy. Program implementation to improve MHHM has focused on access and the hygiene component rather than the health component. The Menstrual Hygiene Management guidelines were developed as part of the Swach Bharat Mission which aims to increase toilets, sanitary pads and disposal facilities within households and schools [29]. These results demonstrate the need to include programs to reduce restrictions around menstruation; increase messaging about maintaining clean cloth since it is a prevalent behavior; ensuring sanitary pads are available within health facilities at a nominal cost and improve access to disposal facilities. Evaluation of the programs provided by the government are also critical to determine if the reach, dose and intensity will meet the national and international goals.

Limitations of this research question include internal validity and causality. Selection could be a threat since the intervention group was purposively chosen based on village characteristics. However, matching on socio-demographics and having a random sample of respondents in both the intervention and comparison villages reduced this risk. Secondly, there might have been participants who dropped out of the program. To decrease this threat, an encoded exposure variable was created which assessed the intervention using dose and recall. The results from this study demonstrated significant differences in socio-demographic/socio-economic characteristics across participants in intervention villages which could affect the results found. The evaluation was cross-sectional in nature which affected causal inferences. Generalizability of these findings are limited to rural India settings.

## Conclusions

The objective of this research was to understand if encoded exposure to a SBCC intervention (*GARIMA),* comprising of dose and recall had an effect on knowledge, attitudes, interpersonal communication, restrictions and MHHM. The results showed that adolescent girls in the ‘high’ encoded exposure group had higher knowledge about puberty, higher knowledge about reproductive parts, positive attitudes towards gender and significantly higher levels of some discussion and dialogue. These mediators also predicted all behaviors corresponding to MHHM. However, the intervention was not successful in addressing knowledge about absorbent use, attitudes towards absorbent use, attitudes towards social/religious restrictions, personal restrictions and structural restrictions, which were significantly associated with some MHHM behaviors. Future programs should use SBCC to improve MHHM behavior but involve families, peers and community members to a greater extent in order to improve attitudes towards menstruation, attitudes towards restrictions, attitudes towards absorbent use and reduce restrictions within the community.

## Data Availability

The dataset used and/or analysed during the current study is available from the corresponding author on reasonable request.

## Appendix 1

Combinations for Categorizing Villages in Intervention and Comparison Blocks

Sampling frame for intervention villages, for each district will be stratified by combining demographic indicators – Village population (no. of households), Proportion of SC-ST population, Distance from District HQ, and presence of educational and health facilities. All five variables are being measured in different units, and hence, difficult to combine. It is therefore essential to convert each one of these into dichotomous variables and create 32 (2^5) different combinations.

It is noteworthy that out of all five variables, three (village population, presence of education and health facility) are in one direction, whereas the other two (% of SC-ST population and Distance from district HQ) are in the opposite direction. Before combining these, we recode them in way that all variables are in the same direction:

1. Village population: High/1 [Above district median]; Low/2 [Below district median]
2. % of SC-ST population: Low/1 [Below district median]; High/2 [Above district median]
3. Distance from district HQ: Low/1 [Below district median]: High/2 [Above district median]
4. Presence of education facility: Presence/1; Absence/2
5. Presence of health facility: Presence/1; Absence/2

For the first three variables, categories have been formed around the respective medians, and hence, will have 50-50 percent of the sampling frame on either side of the median value. For the other two variables, the point of inflection will be actual presence/absence of health and education facility.

Bring together all five dichotomous variables would provide us with 32 different combinations, as provided below:

**Table:**

Cat. No.	Village Size	SC-ST population	Distance from District HQ	Presence: EducationFacility	Presence: Health Facility	Count of 1s	Incidence (in %)
1	1/High	1/Low	1/Low	1/Presence	1/Presence	5	3.125
2	1/High	1/Low	1/Low	1/Presence	2/Absence	4	15.625
3	1/High	1/Low	1/Low	2/Absence	1/Presence	4	
4	1/High	1/Low	2/High	1/Presence	1/Presence	4	
5	1/High	2/High	1/Low	1/Presence	1/Presence	4	
6	2/Low	1/Low	1/Low	1/Presence	1/Presence	4	
7	1/High	1/Low	1/Low	2/Absence	2/Absence	3	31.25
8	1/High	1/Low	2/High	1/Presence	2/Absence	3	
9	1/High	1/Low	2/High	2/Absence	1/Presence	3	
10	1/High	2/High	1/Low	1/Presence	2/Absence	3	
11	1/High	2/High	1/Low	2/Absence	1/Presence	3	
12	1/High	2/High	2/High	1/Presence	1/Presence	3	
13	2/Low	1/Low	1/Low	1/Presence	2/Absence	3	
14	2/Low	1/Low	1/Low	2/Absence	1/Presence	3	
15	2/Low	1/Low	2/High	1/Presence	1/Presence	3	
16	2/Low	2/High	1/Low	1/Presence	1/Presence	3	
17	1/High	1/Low	2/High	2/Absence	2/Absence	2	31.25
18	1/High	2/High	1/Low	2/Absence	2/Absence	2	
19	1/High	2/High	2/High	1/Presence	2/Absence	2	
20	1/High	2/High	2/High	2/Absence	1/Presence	2	
21	2/Low	1/Low	1/Low	2/Absence	2/Absence	2	
22	2/Low	1/Low	2/High	1/Presence	2/Absence	2	
23	2/Low	1/Low	2/High	2/Absence	1/Presence	2	
24	2/Low	2/High	1/Low	1/Presence	2/Absence	2	
25	2/Low	2/High	1/Low	2/Absence	1/Presence	2	
26	2/Low	2/High	2/High	1/Presence	1/Presence	2	
27	1/High	2/High	2/High	2/Absence	2/Absence	1	15.625
28	2/Low	1/Low	2/High	2/Absence	2/Absence	1	
29	2/Low	2/High	1/Low	2/Absence	2/Absence	1	
30	2/Low	2/High	2/High	1/Presence	2/Absence	1	
31	2/Low	2/High	2/High	2/Absence	1/Presence	1	
32	2/Low	2/High	2/High	2/Absence	2/Absence	0	3.125
Total							100.00

On the basis of counts of 1s, we classify 32 different combinations into six broad categories – ‘1’ in all five variables [n=1]; ‘1’ in any four variables [n=5]; ‘1’ in any three variables [n=10]; ‘1’ in any two variables [n=10]; ‘1’ in any one variable [n=5] and zero ‘1’ in five variables [n=1]. We plan to stratify the sampling frame for all intervention and control districts within these six categories, and sample proportionate number of villages from each stratum. For control areas, it will be ensured that similar proportion of villages (as in intervention areas) were sampled from each of the six categories.

## Appendix 2

Creating Combinations for Categorizing Adolescent Girls in Intervention and Comparison Blocks

Listing and main interview data for adolescent girls in control and intervention villages, respectively has four demographic indicators (age, religion, social category, and school-going status), which would be combined, and thereafter be used for matching adolescent girls in control villages. In order to keep the number of combinations to the minimum, all variables would be turned dichotomous in nature. With four variables, we would have a total of 16 (2^4) combinations.

- Age [(A) 12-15 years and (B) 16-19 years];
- Religion [(A) *Hindus* and (B) Rest];
- Social Category [(A) Rest and (B) Scheduled Castes + Scheduled Tribes];
- School Going Status [(A) Yes and (B) No]

Combining four dichotomous variables provides us with sixteen distinct combinations, as listed in the table below. As a result, each girl in intervention villages (main interviews data) and control villages (listing data) would have a new four-character variable ‘Combination’. This variable, as seen below, could have a total of sixteen categories. The matching exercise would ensure that the matched girl in control village has exactly same combination as her counterpart in intervention village.

For example, in intervention villages, a 13-year-old *Hindu*, scheduled caste girl, who does not go to school would be coded as AABB. We would have to find a girl in control villages with the same code (AABB) to ensure both have matching demographics.

**Table:**

Cat. No.	Age	Religion	Social Category	School-Going Status	Combinations
1	A/(12-15 years)	A/*Hindu*	A/ Rest	A/Going to school	AAAA
2	A/(12-15 years)	A/*Hindu*	A/ Rest	B/Not going to school	AAAB
3	A/(12-15 years)	A/*Hindu*	B/SC-ST	A/Going to school	AABA
4	A/(12-15 years)	B/Rest	A/ Rest	A/Going to school	ABAA
5	B/(16-19 years)	A/*Hindu*	A/ Rest	A/Going to school	BAAA
6	A/(12-15 years)	A/*Hindu*	B/SC-ST	B/Not going to school	AABB
7	A/(12-15 years)	B/Rest	A/ Rest	B/Not going to school	ABAB
8	A/(12-15 years)	B/Rest	B/SC-ST	A/Going to school	ABBA
9	B/(16-19 years)	A/*Hindu*	A/ Rest	B/Not going to school	BAAB
10	B/(16-19 years)	A/*Hindu*	B/SC-ST	A/Going to school	BABA
11	B/(16-19 years)	B/Rest	A/ Rest	A/Going to school	BBAA
12	A/(12-15 years)	B/Rest	B/SC-ST	B/Not going to school	ABBB
13	B/(16-19 years)	A/*Hindu*	B/SC-ST	B/Not going to school	BABB
14	B/(16-19 years)	B/Rest	A/ Rest	A/Going to school	BBAB
15	B/(16-19 years)	B/Rest	B/SC-ST	A/Going to school	BBBA
16	B/(16-19 years)	B/Rest	B/SC-ST	B/Not going to school	BBBB

## Abbreviations

95% CI: 95% confidence interval
ad: adequate
AOR: Adjusted odds ratio
Inad: Inadequate
MHHM: Menstrual health and hygiene management
SBCC: Social and behavioral change communication
Semiad: Semi-adequate
